# Immune checkpoint inhibitors in cervical cancer: Current status and research progress

**DOI:** 10.3389/fonc.2022.984896

**Published:** 2022-10-27

**Authors:** Yunkai Xie, Weimin Kong, Xiaoling Zhao, He Zhang, Dan Luo, Shuning Chen

**Affiliations:** Department of Gynecological Oncology, Beijing Obstetrics and Gynecology Hospital, Capital Medical University, Beijing Maternal and Child Health Care Hospital, Beijing, China

**Keywords:** immune checkpoint inhibitors, cervical cancer, PD- 1/L1, immunotherapy, clinical trials

## Abstract

Cervical cancer is the second most common gynecological malignant tumor endangering the health of women worldwide. Despite advances in the therapeutic strategies available to treat cervical cancer, the long-term prognosis of patients with recurrent and metastatic cervical cancer remains unsatisfactory. In recent years, immune checkpoint inhibitors (ICIs) have shown encouraging efficacy in the treatment of cervical cancer. ICIs have been approved for use in both first- and second-line cervical cancer therapies. This review summarizes the current knowledge of ICIs and the application of ICIs in clinical trials for the treatment of cervical cancer.

## 1 Introduction

Cervical cancer is the fourth most common malignant tumor among women in the world in terms of morbidity and mortality (1). According to the latest global cancer data released by the World Health Organization’s International Agency for Research on Cancer (IARC), it is estimated that there were 604,000 new cases of cervical cancer associated with 342,000 deaths in 2020 worldwide. They account for 6.5% and 7.7%, respectively, of new cancer cases and deaths among women worldwide (2).

Generally, the prognosis of early-stage cervical cancer is good, with an overall 5-year survival rate of approximately 70%-90% (3). However, the prognosis of locally advanced cervical cancer and recurrent/metastatic cervical cancer is still poor (4). For patients with locally advanced cervical cancer (FIGO 2018 stages IB3-IVA), concomitant cisplatin-based chemoradiation followed by intrauterine brachytherapy is both recommended by the European Society of Gynecological Oncology (ESGO) and the National Comprehensive Cancer Network (NCCN) (5, 6), but the prognosis is relatively poor, with a 5-year survival rate of approximately 65% (7). The main treatments for patients with recurrent or metastatic cervical cancer are surgery, radiotherapy, and chemotherapy. Unfortunately, the 5-year survival rate for women with recurrent or metastatic cervical cancer is only 16.5% (8, 9).

Breakthroughs in advanced cervical cancer treatments have been slow. It becomes more difficult to further prolong the survival time of patients with radiotherapy and chemotherapy. In recent years, targeted therapy and immunotherapy have emerged to treat recurrent and metastatic cervical cancer. In particular, immune checkpoint inhibitors (ICIs) have greatly improved the survival of patients with cervical cancer in several clinical studies, thus providing new hope for patients with metastatic and recurrent cervical cancer. In this narrative review, we searched the PubMed database, abstracts of major scientific meetings, and clinical trials database (ClinicalTrials.gov) up to July 2022 to provide an overview of the use of ICIs in the treatment of advanced cervical cancer, including its mechanism of action and relevant clinical trials.

## 2 ICIs in cervical cancer

### 2.1 ICIs and cancer immunotherapy

**Figure 1:**
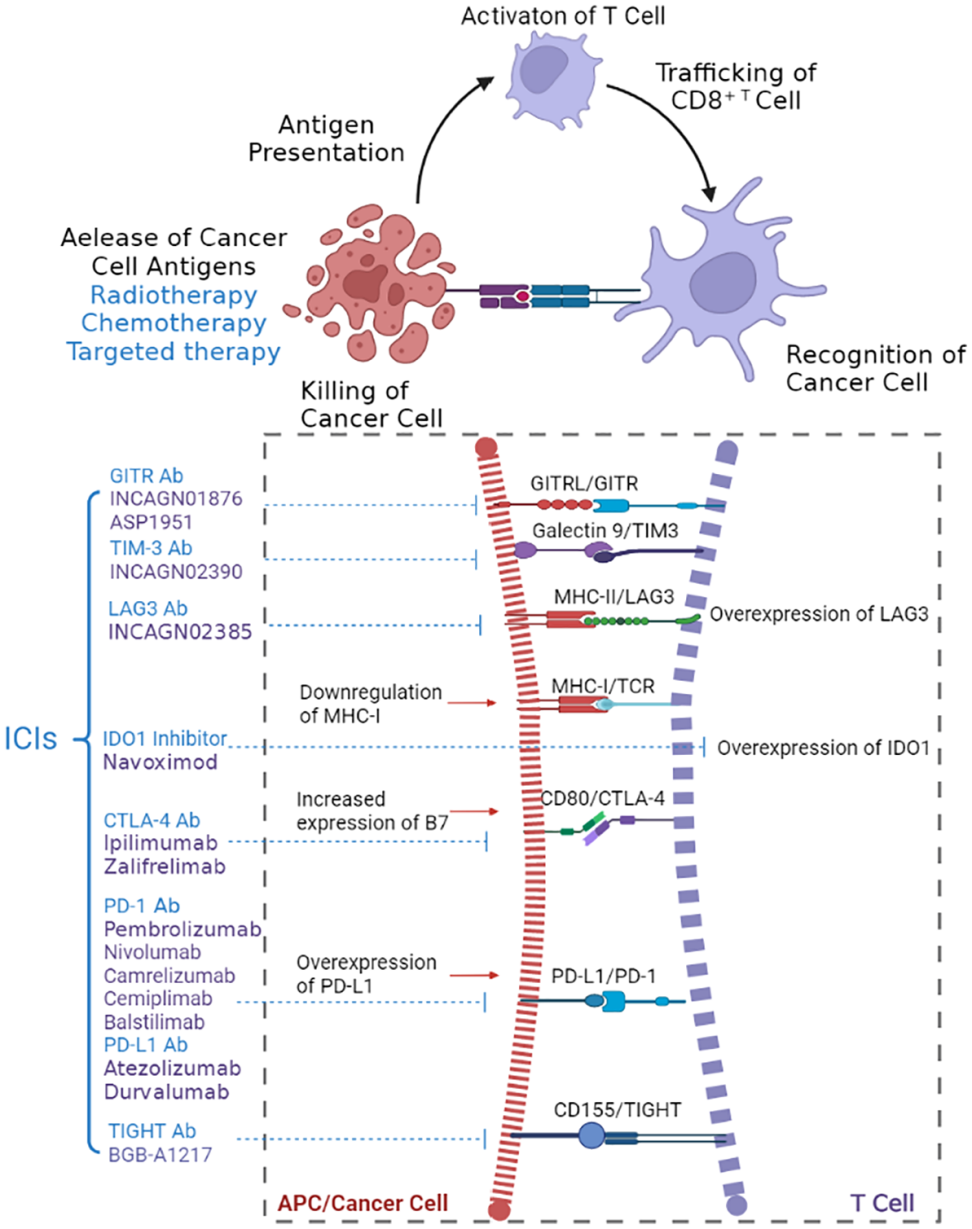
Mechanism of action of ICIs in the anticancer immune response. Created with ^©^ BioRender.

With the advancement of tumor immunology research in the last two decades, immunotherapy has emerged as the most promising anti-cancer strategy. The anticancer immune response can be described as the “cancer immunity cycle” (**Figure 1**) (10). The cancer cell death caused by chemotherapy, radiotherapy, and targeted therapy leads to the release of neoantigens created by oncogenesis, which are presented by antigen presenting cells (APC) to effector T cells. Effector T cells are then activated in lymph nodes and travel to infiltrate the tumor, specifically to recognize and kill their target cancer cell. Immunotherapy can target specific steps in the “cancer immunity cycle” and stimulate the immune system to kill cancer cells.

However, cancer cells develop a variety of mechanisms to evade the identification and eradication of immune cells (11). Immune checkpoints play a key role in the immune escape of tumor cells. Immune checkpoints are immunosuppressive molecules that function as negative regulators to regulate the immune response, which are important pathways for the immune system to avoid self-immune response (12, 13). The most studied immune checkpoints in cervical cancer include programmed death 1/programmed death ligand-1 (PD-1/PD-L1) and cytotoxic T lymphocyte antigen (CTLA-4). PD-1 is a trans-membrane protein expressed on a variety of immune cells, including T cells, B cells, monocytes, natural killer cells, and dendritic cells (11, 14–16). PD-1 in T cells binds to overexpressed PD-L1 in tumor cells, leading to the immunosuppressive activity of T cells (17). CTLA-4 protein is a T cell surface receptor constitutively expressed on regulatory T cells (Tregs) and binds to its ligands (CD80 and CD86). CTLA-4 is believed to inhibit T-cell proliferation by blunting the antigen presenting cells thus providing negative feedback in the immune response (12, 18).Other immune checkpoints being studied in cervical cancer include indoleamine 2,3-dioxygenase 1 (IDO1), lymphocyte-activation gene 3 (LAG3), T-cell immunoglobulin and mucin domain-containing protein 3 (TIM3), glucocorticoid-induced TNFR-related protein (GITR), and T cell immunoreceptor with Ig and ITIM domains (TIGHT). IDO1 is a rate-limiting metabolic enzyme overexpressed in T cells of tumor patients. IDO1 can inhibit the function of CD8+ effector T cells and natural killer (NK) cells in the tumor microenvironment, mediating potent immunosuppressive effects in cancer (19, 20). LAG3 is an inhibitory receptor that is overexpressed on T cells in many types of cancer and can negatively regulate T cell function (21). GITR is a transmembrane protein expressed on Treg, CD4+ and CD8+ T cells, and B cells, inducing responder T cell resistance to Treg-mediated suppression (22).TIM3, and TIGHT are two other inhibitory receptors that are expressed in multiple immune cells and can also regulate the immune response through complex mechanisms (23, 24). TIM3 can mediate inhibition of T cell proliferation, reduction in cytokine production *via* TIM3/Gal9 signaling pathway (25). TIGHT binds to CD155, which expressed on multiple cell types, and induces a tolerogenic phenotype in T cells (24).

Given the role of immune checkpoints in immunosuppression, targeted blockage of immune checkpoint molecules pathway would theoretically enhance the anti-tumor effect of immune cells. ICIs are antibodies targeting immune checkpoint molecules that negatively regulate the anticancer immune response and have been approved as first- or second-line therapies for multiple solid tumors (26, 27). With the advance of clinical research, ICIs have changed the clinical practice of cervical cancer.

### 2.2 Biomarkers for ICI immunotherapy

PD-L1 has been reported to be a new biomarker of cervical cancer, and its expression has been shown to be related to the response to ICI therapy in multiple solid tumors (28, 29). Studies have shown that PD-L1 is overexpressed in cervical intraepithelial neoplasia and cervical cancer but not in normal cervical tissue (30, 31). The PD-L1 expression rates in cervical squamous cell carcinoma (SCC) and cervical adenocarcinoma (ADC) are 55-85% and 64%, respectively (32). In addition, studies have shown a significant association between HPV positivity and PD-L1 expression (31, 33). Liu et al. (33) reported that there was a positive correlation between HPV E7 oncoprotein and PD-L1 expression in cervical cancer. Additionally, PD-L1 can also be widely observed in tumor-invading lymphocytes (TILs) of cervical cancer (34).

Tumor mutation burden (TMB) is a numeric index used to evaluate the number of mutations in a neoplasm (35). As many studies have shown correlations between TMB and response to ICI therapies in multiple tumors (36, 37), TMB has been used as a biomarker to predict the response to immune checkpoint inhibitor treatment (38). In 2020, TMB was approved as a companion diagnostic for the use of pembrolizumab in the treatment of patients with advanced or metastatic solid cancers (39). Data from the KEYNOTE-158 trial showed that the proportion of TMB-H cases for cervical cancer is approximately 15%, and TMB-H cervical cancer tumors showed a trend toward better response rates (36). In turn, McGrail et al. (40) analyzed the data from The Cancer Genome Atlas, which included 21 cervical cancer patients, and obtained a negative outcome. Therefore, the predictive value of TMB in ICI treatment of cervical cancer needs further study.

The DNA mismatch repair (MMR) system is a critical mechanism for identifying and repairing mismatched nucleotides (41). Microsatellites are short, repetitive elements in genomic DNA. The microsatellite repeat number may be altered due to mismatch repair deficiency (dMMR), which is called microsatellite instability (MSI) (42, 43). Deficiency in DNA repair mechanisms results in a hypermutated genome, making it a surrogate marker of TMB, as well as a predictive biomarker for ICI therapy. Patients with MSI/dMMR showed a lasting response to pablizumab in multiple clinical trials (28, 44–46), but the proportion of MSI mutations in cervical cancer is only approximately 3-%-11.3%, as shown in previous studies (47, 48).

In addition, dMMR may lead to mutation of the DNA polymerase gene ϵ/delta 1 (POLE/POLD1) (49), which is another potential biomarker for ICI therapy. Wang et al. (50) analyzed the data from 47,721 patients with multiple cancer types and found that patients with POLE/POLD1 mutations have a significantly longer median overall survival (mOS) than the wild-type population (34 m vs. 18 m log-rank test, χ2 = 8.4; P = 0.004). However, there is no research on the mutation of POLE/POLD1 in cervical cancer. In addition, Qu’s study identified a novel six-gene (APOC1, GLTP, ISG20, SPP1, SLC24A3 and UPP1) signature using bioinformatics technology to predict the response of patients with cervical cancer to ICI therapy (51).

The phenotype of tumor-infiltrating lymphocytes (TILs) was also associated with the ICI therapy response. In a study on T-cell subsets in cervical cancer, Heeren et al. (52) found that activated regulatory T-cell (aTreg) rates were higher in anti-PD-1 nonresponders, while the levels of CD8FoxP3CD25 T cells were elevated in responders.

## 3 Clinical research progress of ICIs in cervical cancer

### 3.1 ICI used in the treatment of cervical cancer

A variety of ICIs have been reported to be used in clinical research of cervical cancer since 2015. These ICIs include anti-PD-1 antibodies [pembrolizumab (53–55), nivolumab (56–58), camrelizumab (59), cemiplimab (60), and balstilimab (61)], anti-PD-L1 antibodies [atezolizumab (62) and durvalumab (63)], anti-CTLA4 antibodies [ipilimumab (64) and zalifrelimab (65)], anti-transforming growth factor-β (TGF-β)/PD-L1 bispecific antibodies [M7824/bintrafusp alfa (66)], IDO1 inhibitors [navoximod (67)] and anti-NKG2A antibodies [monalizumab (68)]. Other novel ICIs that have been registered for clinical research include the anti-PD-1 antibodies tislelizumab (NCT04693234), the anti-GITR antibodies ASP1951 (NCT03799003) and INCAGN01876 (NCT03126110), the anti-TIM-3 antibody INCAGN02390 (NCT03652077), the anti-LAG-3 antibody INCAGN02385 (NCT03538028), and the anti-TIGIT antibody BGB-A1217 (NCT04693234). Among those ICIs, pembrolizumab and nivolumab have been recommended by the NCCN guidelines for the treatment of cervical cancer.

### 3.2 ICI monotherapy in cervical cancer

Several studies have explored the role of ICI monotherapy in recurrent or metastatic cervical cancer, as shown in **Table 1**. However, ICI monotherapy in cervical cancer did not show satisfactory results in most previous studies.

**Table 1:** Trials of ICI single-agent therapy in advanced cervical cancer.

**ICIs**	**Drug**	**Study**	**Population**	**Study phase**	**Treatment**	**Number of Patients (n)**	**Results**
**Anti-PD-1 antibody**	Pembrolizumab	KEYNOTE-158 (53)	Advanced Cervical Cancer	II	pembrolizumab 200 mg/3 weeks	98	ORR = 12.2% (95% CI:6.5%–20.4%); PD-L1(+)subgroup: ORR =14.6%(95% CI:7.8%–24.2%)
		Keynote-028 (54)	Advanced, PD-L1-positive cervical cancer	Ib	pembrolizumab 10 mg/kg/2 weeks	24	ORR = 17% (95% CI:5–37).
		NCT02721732 (55)	Cervical small cell carcinoma	II	pembrolizumab 200 mg/3 weeks	6	ORR = 0 in cervical small cell carcinoma
	Nivolumab	NRG-GY002 (56)	Persistent, recurrent or metastatic cervical cancer	II	nivolumab 3 mg/kg/2 weeks	26	ORR = 4% (90% CI: 0.4–22.9)
		Tamura et al. (57)	Advanced or recurrent cervical cancer	II	nivolumab 240 mg/2 weeks	20	ORR = 25% (80 CI: 13–41); PD-L1(+) group ORR = 33% (80% CI:17–53)
		Checkmate-358 (58)	Recurrent or metastatic cervical cancer	I/II	nivolumab 240 mg/2 weeks	19	mOS = 21.9 m (95% CI, 15.1 m- not reached) ORR = 26.3% (95% CI: 9.1–51.2)
	Cemiplimab	Rischin et al. (60)	Recurrent or metastatic cervical cancer	I	Cemiplimab 3 mg/kg/2 weeks or Cemiplimab +hfRT	10/10	Cemiplimab group: ORR = 10% (95% CI: 0.3%–44.5%)
		EMPOWER-Cervical 1/GOG-3016/ENGOT-cx9 (69)	Disease progression after first-line platinum-containing chemotherapy	III	Cemiplimab 350 mg/3 weeks or chemotherapy	608	ORR = 16.4% (95% CI,12.5%-21.1%)
	Balstilimab	David M O'Malley et al. (61)	Recurrent or metastatic cervical cancer	II	Balstilimab 3 mg/kg/week	140	ORR = 15% (95% CI: 10.0–21.8); PD-L1(+)group ORR = 20%.
**Anti-CTLA-4 antibody**	Ipilimumab	Lhereux et al. (70)	Recurrent or metastatic cervical cancer	I/II	ipilimumab, 10 mg/kg/3 weeks for 4 cycles	34	PFS= 2.5 m (2.1 m-3.2 m); OS = 8.5 m (3.6 m--not reached); ORR= 2.94%
		NCT01693783	Recurrent or metastatic cervical cancer	II	Ipilimumab	–	Underway
**Anti-CD94/NKG2Abispecific antibody**	Monolizumab	Anna V Tinker et al. (68)	Advanced, recurrent or metastatic gynecologic cancers	I	monalizumab 10 mg/kg/2 weeks	9	ORR = 0%
**Anti-TGF-ß/PD-L1 bispecific antibody**	Bintrafusp alfa	NCT02517398, NCT03427411 (66)	Metastatic or Locally Advanced Solid Tumors	I/II	bintrafusp alfa/2 weeks	33	ORR = 30%
		NCT04246489	Platinum-experienced cervical cancer	II	bintrafusp alfa	146	Underway
**Anti-TIM-3 antibody**	INCAGN02390	NCT03652077	Locally advanced or metastatic cervical cancer	I	INCAGN02390	40	Completed
**Anti-LAG-3 antibody**	INCAGN02385	NCT03538028	Locally advanced or metastatic cervical cancer	I	INCAGN02385	22	Completed
**Anti-GITR antibody**	INCAGN01876	NCT03126110	Advanced or metastatic cervical cancer	I/II	INCAGN01876	145	Completed
**Anti-CTLA-4 x LAG-3 bispecific antibody**	XmAb22841	NCT03849469	Advanced cervical cancer	I	XmAb22841	242	Underway

KEYNOTE-158 is a phase II basket study that included 98 patients with advanced cervical cancer. Patients in the study received 3 weekly doses of pembrolizumab 200 mg for up to 2 years. All responses were only seen in patients with PD-L1-positive tumors, for an overall response rate (ORR) of 14.6% (95% CI, 7.8% to 24.2%) (53). In the Checkmate-358 study, nivolumab showed an ORR of 26.3% (95% CI: 9.1%–51.2%) in patients with advanced cervical cancer (58). Another phase II study reported by Kenji Tamura showed that the ORR of nivolumab in advanced cervical cancer patients with PD-L1-positive tumors was 33% (80% CI: 17%–53%) (57).Based on the results of the above studies, the NCCN guidelines recommended pembrolizumab as a second-line regimen indicated for recurrent PD-L1-positive or MSI-H/dMMR cervical cancer in 2020 (71) and added nivolumab as the second-line therapy for PD-L1-positive cervical cancer in 2022 (5).

The latest research (EMPOWER-Cervical 1/GOG-3016/ENGOT-cx9) published in the New England Journal of Medicine is the only and largest phase III clinical trial on ICI monotherapy in cervical cancer (69). A total of 608 patients with advanced cervical cancer were included in this study and received either cemiplimab 350 mg every 3 weeks or chemotherapy (pemetrexed, vinorelbine, gemcitabine, irinotecan or topotecan). The results showed that in the overall trial population, mOS was longer in the cemiplimab group than in the chemotherapy group (12.0 m vs. 8.5 m; hazard ratio for death: 0.69; 95% CI: 0.56–0.84; P<0.001). The ORR of the patients in the cemiplimab group was 16.4% (95% CI, 12.5%– 21.1%), compared with 6.3% (95% CI, 3.8%-9.6%) in the chemotherapy group (69). Another phase II clinical trial evaluated the antitumor activity of balstilimab in patients with recurrent/metastatic cervical cancer. This study included 140 patients, and the ORR was 15% (95% CI, 10.0%-21.8%) (61). These studies showed that cemiplimab may be another promising ICI for the treatment of cervical cancer.

It is worth noting that some monotherapy with bispecific antibodies has shown a better effect in cervical cancer. An analysis of data from two trials (NCT02517398/NCT03427411) provided encouraging outcomes for the use of bintrafusp alfa (anti-TGF-ß/PD-L1 bispecific antibody) in advanced cervical cancer, with 30% (10/33) of patients having a clinical response (66). A larger clinical trial (NCT04246489) of bintrafusp alfa monotherapy in patients with advanced, unresectable cervical cancer is underway. Furthermore, some completed studies (NCT03652077, NCT03538028, NCT03126110) about other novel ICIs may provide more encouraging results.

### 3.3 Combinations of different ICIs: Are two better than one

The anticancer immune response is a complex physiological process, which involves antigen presentation, activation of T cells, and recognition of cancer cells (10). Various types of immune checkpoints may mediate immunosuppression through different and complementary pathways, which may result in low response rates to ICI monotherapy. Virous ICIs and immunotherapy play a role in the steps of the “cancer immunity cycle” because of their different targets. Therefore, the combined use of multiple ICIs and ICIs combined with other immunotherapies can theoretically further exert the antitumor effect of immunotherapy. A number of clinical trials have been conducted to evaluate the efficacy of two ICI combinations and ICIs combined with other immunotherapies in the treatment of cervical cancer (**Table 2**).

**Table 2:** Trials with combined use of two ICIs or ICIs with another immunotherapy.

Study	Drug(s)/Treatment		Population	Study phase	Number of cervical cancer patients (all patients) (n)	Results
NCT03444376 (72)	Anti-PD-1 Antibody	Pembrolizumab	Advanced or metastatic HPV-positive cervical cancer	II	26	ORR = 42% (95% CI:23%–63%) PD-L1 (+) group: ORR = 50% (95% CI:27%–73%)
	DNA vaccine	GX-188E				
NCT03108495	Anti-PD-1 antibody	Pembrolizumab	Recurrent, metastatic, or persistent cervical cancer	II	138	Underway
	TILs	LN-145				
GDC-0919 (67)	TILs	TILs	Locally advanced or metastatic cervical cancer	I	6 (158)	Dose Escalation (n=66) ORR = 9%; Dose Expansion (n=92): ORR = 11%;result in cervical cancer subgroup not mentioned
	IDO1 inhibitor	Navoximod				
Checkmate-358 (73)	Anti-PD-1 Antibody	Nivolumab	Recurrent or metastatic cervical cancer	I/II	91	NIVO3+IPI1group (n=45): ORR=26.7%; NIVO1+IPI3group (n=46): ORR=41.3%; Overall ORR=34.1%
	Anti-CTLA-4 Antibody	Ipilimumab				
C550 (74)	Anti-PD-1 Antibody	Balstilimab	Metastatic or locally advanced cervical cancer	II	125	ORR=25.6% (95% CI,18.8-33.9); mOS=12.8 m(95% CI:8.8-17.6); PD-L1(+) group: ORR=32.8%, mOS=15.7 m(95% CI:7.6-21.1)
	Anti-CTLA-4 Antibody	Zalifrelimab				
Yin et al. (75)	Anti-PD-1 antibody	Nivolumab	Persistent, recurrent, or metastatic cervical cancer	I	80	ORR= 25%
	TILs	TILs				
NCT04300647	Anti-TIGIT Antibody	Tiragolumab	Metastatic and/or Recurrent PD-L1-Positive Cervical Cancer	II/III	172	Underway
	Anti-PD-L1 antibody	Atezolizumab				
NCT04693234	Anti-PD-1 Antibody	Tislelizumab	Recurrent or metastatic cervical cancer	II	167	Underway
	Anti-TIGIT Antibody	BGB-A1217				
NCT03799003	Anti-GITR antibody	ASP1951	Locally-advanced or metastatic cervical cancer	I	120	Underway
	Anti-PD-1 antibody	Pembrolizumab				
NCT04380805	Anti-PD-1/CTLA-4 bispecific antibody	AK104	Recurrent or metastatic cervical cancer	II	–	Underway
NCT05063916			Recurrent Small cell neuroendocrine carcinomas of the cervix	II	–	Underway
NCT05235516		AK104+ ratio therapy	Locally advanced cervical cancer	III	–	Underway
NCT04982237		AK104+ Paclitaxel + Cisplatin± Bevacizumab	Persistent, recurrent, or metastatic cervical cancer	III	–	Underway
NCT04868708		AK104+ Paclitaxel + Cisplatin	Recurrent or metastatic cervical cancer	II	–	Underway

The C550 study is the largest reported phase II study that evaluated the antitumor activity of the combination of balstilimab (an anti-PD-1 antibody) with zalifrelimab (an anti-CTLA-4 antibody) as a second-line therapy for advanced cervical cancer. A total of 125 patients with recurrent or metastatic cervical cancer were included and were followed up for 21 months. The confirmed ORR was 25.6% (95% CI, 18.8%-33.9%), with an ORR of 32.8% in patients with PD-L1-positive tumors (74). The Checkmate-358 study evaluated the effectiveness of the combination of nivolumab with ipilimumab. A total of 91 patients with recurrent or metastatic cervical cancer were randomized 1:1 to either nivolumab (3 mg/kg/2 weeks) + ipilimumab (1 mg/kg/6 weeks) (nivo3 + ipi1) or nivolumab (1 mg/kg/2 weeks) + ipilimumab (3 mg/kg/3 weeks for 4 doses) followed by nivolumab (240 mg/2 weeks) (nivo1 + ipi3), and obtained an ORR of 34.1% for all populations (73). The above studies suggest that double ICIs may be a promising therapy for advanced cervical cancer. Some studies (NCT04693234, NCT03799003) on the combination of anti-PD-1 and anti-TIGHT antibodies or with anti-GITR antibody are underway. At the same time, some dual ICIs, such as AK-104 (anti-PD-1/CTLA-4 bispecific antibody), have been registered for clinical research on cervical cancer (NCT04380805; NCT05063916; NCT05235516; NCT04982237; NCT04868708).

Other studies have evaluated the antitumor activity of ICIs combined with another immunotherapy in cervical cancer, including therapeutic cancer vaccines and tumor-infiltrating lymphocyte (TIL) therapy. The interim results of a phase II clinical study published in Lancet Oncology revealed the efficacy of ICIs combined with a therapeutic cervical cancer vaccine in advanced cervical cancer. Twenty-six patients in that study received seven doses of the 2 mg GX-188Evaccine (a therapeutic HPV DNA vaccine) in addition to pembrolizumab 200 mg every 3 weeks and were evaluable for interim activity assessment. The results showed that 11 of 26 patients achieved an overall response (ORR=42%; 95% CI 23-63) (72). TIL therapy is an adoptive cell therapy (adoptive cell therapy, ACT), in which tumor-infiltrating lymphocytes are isolated, cultured and expanded and then injected into patients to exert their antitumor activity (76). A phase I study investigated the efficacy of TIL and anti-PD1 combination therapy in patients with advanced cervical cancer. An ORR was observed in 20 (25.0%) out of 80 patients with PD-L1-negative and low MSI expression tumors (75). Further phase II studies are needed to evaluate the efficacy and safety of TIL (LN-145) and pembrolizumab combination therapy in patients with advanced cervical carcinoma. These studies are still in their infancy, but they have provided hope for the application of immunotherapy in cervical cancer. In most studies, combination therapy showed a better anti-tumor activity, but whether the combination therapy will cause more treatment emergent adverse events (TEAEs) remains to be further studied.

### 3.4 Combinations of ICIs with chemotherapy, antiangiogenic therapy, radiotherapy, and targeted therapy: The future trend

In view of the unsatisfactory response rate of ICI monotherapy, ICIs are usually used in combination with other therapies. The combinations of ICIs with radiotherapy, chemotherapy, antiangiogenic therapy, and targeted therapy have improved the ORR of cervical cancer in a number of clinical trials (**Table 3**).

**Table 3:** Trials combining the use of ICIs with radiotherapy, chemotherapy, antiangiogenic therapy, and targeted therapy.

Drug		Study	Population	Study phase	Treatment	Number of Patients (n)	Result
**Anti-PD-L1 Antibody**	Atezolizumab	BEATcc study (NCT03556839) (77)	Metastatic (stage IVB), persistent, or recurrent cervical cancer	III	Cisplatin + Paclitaxel + Bevacizumab + Atezolizumab	404	Underway
		NCT03612791	Locally advanced cervical cancer	II	Atezolizumab + CCRT	–	Underway
		NCT02921269 (62)	Metastatic, persistent, or recurrent cervical cancer	II	Bevacizumab + Atezolizumab	11	ORR=0%
	Durvalumab	CALLA study (63)	Locally advanced cervical cancer	III	CCRT + Durvalumab/placebo	–	Underway
**Anti-PD-1 Antibody**	Pembrolizumab	Keynote-826 (78)	Metastatic, persistent, or recurrent cervical cancer	III	Paclitaxel + Platinum±Bevacizumab + Pembrolizumab/placebo	617	Pembrolizumab group (n=308) vs. placebo group (n=309): PFS:10.4 vs. 8.2
		NCT02635360	Advanced cervical cancer	II	CCRT + Pembrolizumab	88	Underway
		NCT03144466	Stage 1B - IVA cervical cancer	I	CCRT + Pembrolizumab	–	Underway
		MK-3475-A18/KEYNOTE-A18/ENGOT-cx11/GOG-3047 (NCT04221945)	Locally advanced cervical cancer	III	Pembrolizumab + CCRT	980	Underway
		PRIMMO study (NCT03192059)	Advanced or refractory cervical cancer	II	Pembrolizumab + Radiation + immunomodulatory cocktail (Vitamin D, aspirin, Cyclophosphamide and Lansoprazole)	43	Completed
		NCT04641728	Recurrent cervical cancer	II	Pembrolizumab + Olaparib	–	Underway
		NCT04865887	Locally advanced or metastatic cervical cancer	II	Pembrolizumab + Lenvatinib	–	Underway
		NCT04238988	Locally advanced cervical cancer	II	Pembrolizumab + Carboplatin+Taxol	–	Underway
	Cemiplimab	Rischin et al. (60)	Recurrent or metastatic cervical cancer	I	Cemiplimab/Cemiplimab + hfRT	10/10	hfRT group: ORR = 10% (95% CI:0.3–44.5)
	Nivolumab	NCT03298893 (79)	Locally advanced cervical cancers	I/II	Nivolumab + CCRT	16	One-year PFS was 81.2% [95% CI: 64.2%-100%].
		GOTIC-018 (80)	Locally advanced cervical cancers	I	Nivolumab + CCRT	30	Cohort A: CR=73.3%; PR=26.7%; Cohort B: CR=66.7%; PR=33.3%
	Toripalimab	ChiCTR2000032879 (81)	Locally advanced cervical cancers	–	Toripalimab +CCRT	22	In 10 m: ORR was 100%
		NCT05084677	Locally advanced cervical cancer	II	Toripalimab + CCRT	96	Underway
	Camrelizumab	CLAP study (59)	Recurrent or metastatic cervical cancer	II	Apatinib + Camrelizumab	45	ORR = 55.6% (95% CI:40.0–70.4); PD-L1+=69%
		NCT05290935	Recurrent or persistent advanced cervical cancer	II	Camrelizumab + Albumin-bound paclitaxel	–	Underway
		NCT04974944	Metastatic, persistent, or recurrent cervical cancer	II	Camrelizumab + Apatinib	–	Underway
	Tislelizumab	NCT05310383	Recurrent cervical cancer	II	Tislelizumab + Radiotherapy	–	Underway
	TSR-042	NCT03833479	Locally advanced cervical cancer after chemo-radiation	II	CCRT + TSR-042	–	Underway
	BCD-100	NCT03912415	Advanced cervical cancer	III	Paclitaxel + Cisplatin/Carboplatin + Bevacizumab + BCD-100	–	Underway
**Anti-CTLA-4 Antibody**	Ipilimumab	GOG-9929(NCT01711515)	Stages IB2-IIB or IIIB-IVA cervical cancer	I	Ipilimumab + CCRT	34	Completed

#### 3.4.1 ICI + chemotherapy

The combinations of ICIs with chemotherapy may enhance the anti-cancer activity of ICIs in two ways. First, chemotherapies may stimulate the immune system by triggering antigen release *via* cytotoxic cell death activity (82). In addition, chemotherapies may impact the activation status of immunocytes in the tumor microenvironment (83). In published clinical trials, the combination of ICI with chemotherapy has shown better anti-tumor activity than chemotherapy alone.The Keynote-826 study (NCT03635567) is the only published phase III clinical study of ICIs combined with chemotherapy and anti-angiogenesis in cervical cancer. The results were published in the New England Journal of Medicine in 2021. A total of 617 patients with persistent, recurrent, or metastatic cervical cancer were randomly assigned in a 1:1 ratio to receive pembrolizumab (n=308) or placebo (n=309) plus platinum-based chemotherapy and, per investigator discretion, bevacizumab. Among 548 patients with a PD-L1 combined positive score (CPS) ≥1, the OS was significantly longer in the pembrolizumab group than in the placebo group (24-month estimate of patients alive, 53.0% [95% CI, 46.0 to 59.4] vs. 41.7% [95% CI, 34.9 to 48.2]; hazard ratio for death, 0.64; 95% CI, 0.50 to 0.81; P<0.001), and the mPFS was 10.4 months in the pembrolizumab group and 8.2 months in the placebo group (78). Based on the exciting results of this study, the NCCN guideline (2022. V1) recommended pembrolizumab + chemotherapy, with or without bevacizumab as the first-line therapy for patients with PD-L1-positive metastatic, persistent, and recurrent cervical cancer (5). Another large phase III clinical study (BEATcc study) evaluating the efficacy of atezolizumab combined with cisplatin-paclitaxel plus bevacizumab in the treatment of cervical cancer is underway (77). In the second-line treatment of cervical cancer, camrelizumab combined with albumin-bound paclitaxel has been used in a study of recurrent or persistent advanced cervical cancer patients after first-line chemotherapy (NCT05290935).

#### 3.4.2 ICI + radiotherapy

Similar to the effects of chemotherapy, radiotherapy turns the immunologically ‘cold’ tumors ‘hot’ and enhances the anti-tumor response of the immune system by triggering the release of neoantigens and increasing tumor-infiltrating immunostimulatory cells (84, 85). However, further large-scale studies of ICIs combined with radiotherapies for cervical cancer are still needed. Rischin et al. (60) reported phase I clinical trials that evaluated the antitumor activity of cemiplimab as a monotherapy or in combination with hypofractionated radiation therapy (hfRT) in patients with recurrent or metastatic cervical cancer, with an ORR of 10% for 10 patients in the hfRT group. Other than that, no clinical study results have been reported for the combination of ICIs with radiotherapy in advanced cervical cancer. However, there are several registered studies evaluating the efficacy of ICIs + concurrent chemoradiotherapy (CCRT) or ICIs + radiotherapy in advanced cervical cancer (NCT03612791, CALLA study, NCT02635360, NCT03144466, MK-3475-A18/KEYNOTE-A18/ENGOT-cx11/GOG-3047, NCT03298893, NCT05310383, NCT03833479, NCT01711515) (**Table 3**).

The interim results of several studies have been reported on the 2022 American Society of Clinical Oncology (ASCO) annual meeting to assess the efficacy and safety of the combination of ICIs with concurrent chemoradiation (CCRT) in patients with locally advanced cervical cancer (GOTIC-018, ChiCTR2000032879, NCT03298893) (79–81). In the NiCOL phase I trial, nivolumab+CCRT showed good efficacy in locally advanced cervical cancer, with a one-year PFS of 81.2% (95% CI: 64.2%-100%) (79). A trial carried out in China showed that toripalimab + CCRT has promising antitumor activity in patients with locally advanced cervical cancer, and the ORR was 100% for 22 patients during the ten-month follow-up period (81). Meanwhile, a phase II study of toripalimab combined with CCRT in locally advanced cervical cancer is underway (NCT05084677). With the release of the research results, ICIs combined with CCRT might represent a novel treatment option for patients with locally advanced cervical cancer.

#### 3.4.3 ICI + antiangiogenic therapy

Although many studies have reported that antiangiogenic drugs can improve the efficacy of ICIs by enhancing antigen presentation, activating cytotoxic CD8 T cells, and promoting lymphocyte infiltration into tumors (86–89).The clinical efficacy of antiangiogenic therapy combined with ICIs in the treatment of cervical cancer remains to be verified. In the reported phase II studies, atezolizumab combined with bevacizumab did not show antitumor activity in advanced cervical cancer (62). However, camrelizumab + apatinib showed satisfactory results in a multicenter, open-label, single-arm, phase II study (CLAP study), with an ORR of 55.6% (95% CI: 40.0–70.4) (59). In some ongoing studies, ICIS plus poly ADP-ribose polymerase (PARP) inhibitors have been used as the second-line treatment of advanced cervical cancer (NCT04641728).

## 4 Conclusion

In recent years, a large number of clinical studies have been carried out to evaluate the safety and effectiveness of immunotherapy in cervical cancer. There is no doubt that ICIs have made a breakthrough in the treatment of advanced metastatic and recurrent cervical cancer. ICIs such as pablizumab have been used in many clinical studies and have achieved encouraging results. With the publication of these clinical studies, the NCCN guidelines recommended pembrolizumab plus chemotherapy, with or without bevacizumab as the first-line therapy for patients with PD-L1-positive advanced cervical cancer, and nivolumab as the second-line therapy for PD-L1-positive cervical cancer. However, how to improve the response rate of cervical cancer to ICI therapy is still a problem worthy of further exploration. According to the existing research results, the combination of ICIs with radiotherapy, chemotherapy and other targeted drugs seems to be an effective treatment. Furthermore, we still need a more accurate model, including the biomarker status and clinical characteristics of patients, to predict the response of cervical cancer to ICIs.

## Author contributions

YX and WK contributed to the conception of the study and wrote the manuscript. WK contributed. All authors contributed to the article and approved the submitted version.

## Conflict of interest

The authors declare that the research was conducted in the absence of any commercial or financial relationships that could be construed as a potential conflict of interest.

## Publisher's note

All claims expressed in this article are solely those of the authors and do not necessarily represent those of their affiliated organizations, or those of the publisher, the editors and the reviewers. Any product that may be evaluated in this article, or claim that may be made by its manufacturer, is not guaranteed or endorsed by the publisher.
